# Endobronchial ultrasound: A new innovation in bronchoscopy

**DOI:** 10.4103/0970-2113.45199

**Published:** 2009

**Authors:** Balamugesh T., Herth F. J.

**Affiliations:** *Department of Pulmonary Medicine, Christian Medical College, Vellore, India*; 1 *Department of Internal Medicine, Pneumology and Critical Care Medicine, Thoraxklinik, University of Heidelberg, Heidelberg, Germany*

**Keywords:** Bronchoscopy, lung cancer, ultrasound

## Abstract

Technical development in last two decades has made it possible for pulmonologists to do endobronchial ultrasound (EBUS). With EBUS mini-probe, the multilayered structure of the tracheobronchial wall can be analyzed better than any other imaging modality. Instead of fluoroscopic guided biopsy, EBUS can be used to biopsy peripheral lesions. EBUS-transbronchial needle aspiration has proved valuable for mediastinal lymph node staging of lung cancer. Studies have shown that EBUS is cost-effective as it reduces the need for more morbid and costly invasive procedure like mediastinoscopy or thoracotomy. Prospective studies are needed in India to see how EBUS will help in populations with high prevalence of tuberculosis.

## INTRODUCTION

Bronchoscopy has become the most commonly performed invasive procedure by pulmonologists. In India fiberoptic bronchoscopy has been performed since 1980s.^1^ Since then it has been increasingly employed in the diagnosis of variety of pulmonary diseases. A survey of bronchoscopic practice in India in 2005 shows that compared to 1997 there is increasing use of videobronchoscopy and interventional procedures like balloon dilatation, brachytherapy, electrocautery and stent insertion.^2^ However none of the respondents in the above survey from India had performed Endobronchial ultrasound (EBUS) although it has been in vogue for the last one decade elsewhere in the world. In this article an attempt has been made to review the application of EBUS based on recent medial literature.

## HISTORY

Computerised Tomography (CT) scan was and continues to be the imaging modality of choice for diagnosis and staging of lung cancer. However CT proved unsuccessful in evaluation of lymph node involvement and for diagnosis of air wall infiltration. Positron Emission Tomography-CT (PET-CT) to a certain extent improved the mediastinal lymph node staging. However to obtain tissue for histopathology, mediastinoscopy or thoracotomy is needed. A better imaging modality was increasingly felt necessary for lung cancer staging. In the late 1980s endoscopic ultrasound (EUS) was introduced in gastroenterology.^3^ However the same could not be used for all mediastinal structures due to interference of air, since air cannot conduct ultrasound waves. After years of painful research it was in 1990s that a dedicated Endobronchial ultrasound system with balloon catheter was made commercially available. Initially it was not widely accepted. The topic of discussion in the conferences used to be “Endobronchial ultrasound – Expensive toy or useful tool?”^4^

However in the past decade increasing number of researchers have found EBUS immensely useful in numerous conditions. EBUS has become a hot topic in all major conferences and is becoming an essential bronchoscopic tool. With the recent introduction of dedicated ultrasonic bronchoscope with real time guidance for needle aspiration there is widespread acceptance of this novel method leading to a ‘new dawn for respiratory physician’.^5^

## TYPES OF EBUS

**Figure 1:**
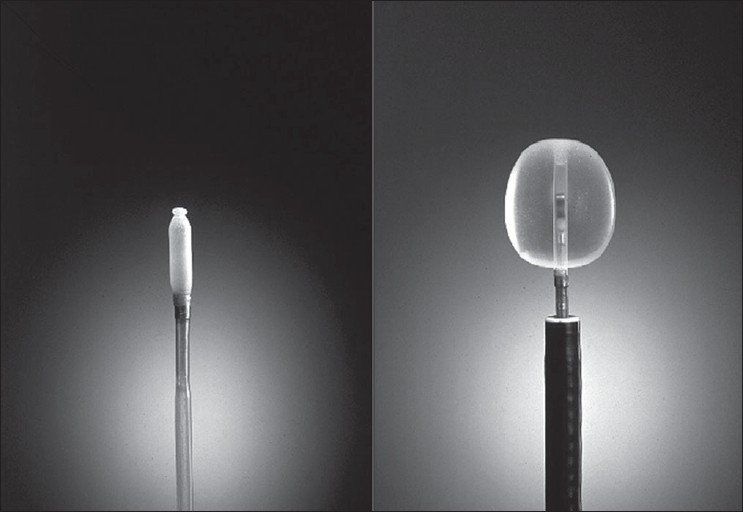
Radial probe EBUS

There are two forms of EBUS, radial and linear (convex). The major differences are given in Table 1.^6^ Both these forms have a transducer and a processor. Transducer produces and receives the sound waves. Processor integrates the reflected sound waves. Depending on the absorption and scatter of the tissues and their interfaces, processor generates ultrasound images. The probe is provided with a balloon which if required can be inflated with water to improve the image by securing good contact with the airways. The radial probe can be introduced through the working channel of a conventional bronchoscope [Figure 1]. The probe if used meticulously can be reused for up to 75 examinations.^6^ The probe is used to visualize the lesion and then withdrawn. A sheath can be left in situ to localize and stabilize the lesion during the subsequent introduction of forceps or brush for sampling. The EBUS-TBNA (transbronchial needle aspiration) bronchoscope using the linear probe has an outer diameter of 6.9 mm. Also the view is 30° from the horizontal and the scopist needs to compensate accordingly. Complete visual inspection of the bronchial tree will not be possible with this EBUS bronchoscope and standard bronchoscopy needs to be performed separately.

**Table 1:** Comparison of the two types of EBUS

	**Radial probe EBUS**	**Linear probe EBUS**
Transducer	Rotating mechanical transducer	Fixed array of electronic transducer aligned in a curvilinear pattern
View	360° to the long axis of scope	60° parallel to the long axis of the scope
Frequency	20 MHz (12, 30 also available)	5–12 MHz
Tissue penetration	4–5 cm	5 cm
Image quality	Very good. Allows airway layers to be identified	Currently not possible to identify airway layers
Real time TBNA	Not possible	Possible
Doppler to indentify blood vessels	Not possible	Possible

## INDICATIONS FOR EBUS

### 1. Access the extent of airway invasion

**Figure 2:**
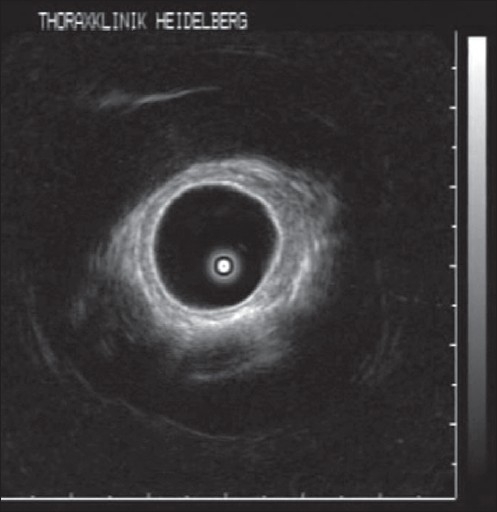
Radial probe EBUS showing multilayered bronchial wall and early invasive cancer which does not invade the cartilage

The view of the bronchoscopist is limited to the lumen and the bronchial mucosa. EBUS has extended the vision beyond the tracheobronchial wall. One of the first indications to use EBUS was for preoperative assessment of airway wall in patients with centrally localized lung tumor. CT scan often cannot predict the extent of tumor invasion into the airway wall. For planning Endobronchial treatment modalities it is important to know if the tumor is extending into or beyond the cartilage or just limited inside the submucosal layers [Figure 2]. This is especially important in differentiating early from invasive lung cancer. In a prospective study, EBUS was found to be superior to CT scan in showing airway involvement by lung cancers.^7^ In differentiating external compression of airways from actual tumor infiltration EBUS has a specificity of 100%, a sensitivity of 89%, and an accuracy of 94% compared to CT, which is far inferior, with a specificity of 28%, a sensitivity of 75%, and an accuracy of 51%. It has been recently confirmed by radial EBUS that normal central airways has 7 layers.^8^ The inner 2 layers in EBUS correspond to mucosa and submucosa, the third, forth, and fifth layer to the cartilage, and the external 2 layers to loose and dense fibroelastic connective tissue surrounding the airway. Beyond the lobar bronchi, external 2 layers progressively taper and thin away and finally when the cartilages become patchy and disappear only 3 layers could be detected.

In advanced tumors the longitudinal extent and patency of distal airways can be assessed by passing the radial probe beyond high-grade stenosis.^9^,^10^ This is of immense value in deciding interventional procedures by helping to measure the length of stenosis and to assess proximity of a blood vessel to the bronchial wall. In fact EBUS was able to guide or modify therapy in 43% of the cases undergoing therapeutic bronchoscopy.^10^,^11^

### 2. Peripheral intrapulmonary lesions

**Figure 3:**
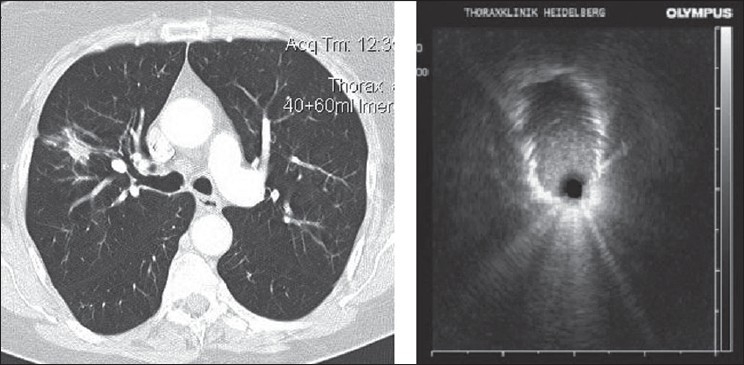
Radial probe EBUS showing the peripheral pulmonary nodule as a hypoechoic structure

Fluoroscopy is generally used during bronchoscopy to guide sampling peripheral pulmonary lesions. CT guidance for peripheral lesions can be considered in prior unsuccessful bronchoscopy but is expensive and has significant radiation exposure.^12^ Radial probe EBUS can be used to localize peripheral pulmonary nodules and sampling of the lesion can be done without fluoroscopy^13^ [Figure 3]. The pulmonary masses have a hypoechoic texture as compared to the surrounding lung and have sharply defined borders due to strong reflective interface produced between the aerated lung and the lesion. In a randomized trail comparing standard bronchoscopic biopsy under fluoroscopy with radial probe EBUS, the yield were 52% and 76% respectively.^14^ EBUS can be even used to sample lesions, which are less than 3 cm in diameter, which are often not seen fluoroscopically. In a study of 54 patients with fluoroscopically invisible Solitary Pulmonary Nodule (SPN) having a mean diameter of 2.2cm, sheathed radial probe EBUS localized the lesion in 48 patients (89%) and yielded a diagnosis in 38 patients (70%).^15^ The diagnosis in nine patients (17%) prevented the patients from undergoing a surgical procedure. The only complication was pneumothorax in one patient.

### 3. Analysis of mediastinal lesions

Assessment of mediastinal lymph nodes is important for lung cancer staging and planning appropriate treatment strategy. Current imaging modalities like CT and PET can indicate size and metabolic activity, respectively but their accuracy is far from satisfactory. American College of Chest Physicians (ACCP) guidelines of 2007 concluded that for mediastinal lymph node metastasis sensitivity and specificity are 51 & 85% for CT and 75% & 85% for PET.^16^ With either imaging abnormal lymph nodes must be confirmed by tissue biopsy, most commonly by mediastinoscopy to ensure accurate staging. But mediastinoscopy is an invasive procedure that requires general anesthesia and is associated with small morbidity and mortality.^17^ TBNA has been found useful in the analysis of mediastinal lymph nodes with high specificity. However, the sensitivity of TBNA in identifying mediastinal lymph node metastasis varies from 39% to 78% depending on the study population.^18^,^19^ Surveys indicate that only 10–30% of pulmonologists regularly use TBNA. One of the reasons for the limited use of TBNA is that it is a blind procedure.^19^,^20^

Radial probe EBUS can be used to localize the mediastinal lymph nodes. Once target lymph node is identified, the probe is removed and a needle is inserted for sampling through the working channel of bronchoscope without real-time needle monitoring. A randomized trial between conventional TBNA and TBNA after EBUS localization, for mediastinal staging demonstrated that EBUS guidance significantly increased the yield of TBNA in all stations (84 versus 58%), except for the subcarinal region,^21^ with out an on-site cytologist.

**Figure 4:**
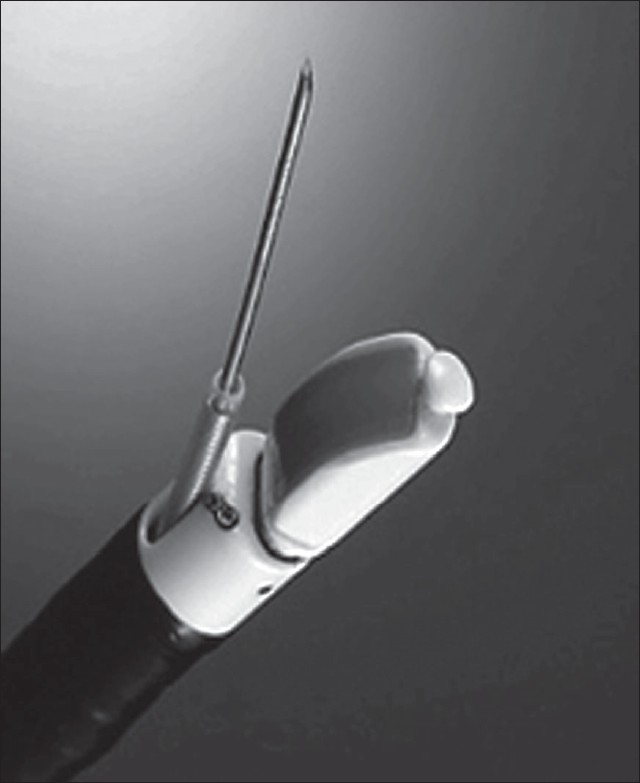
Linear EBUS

**Figure 5:**
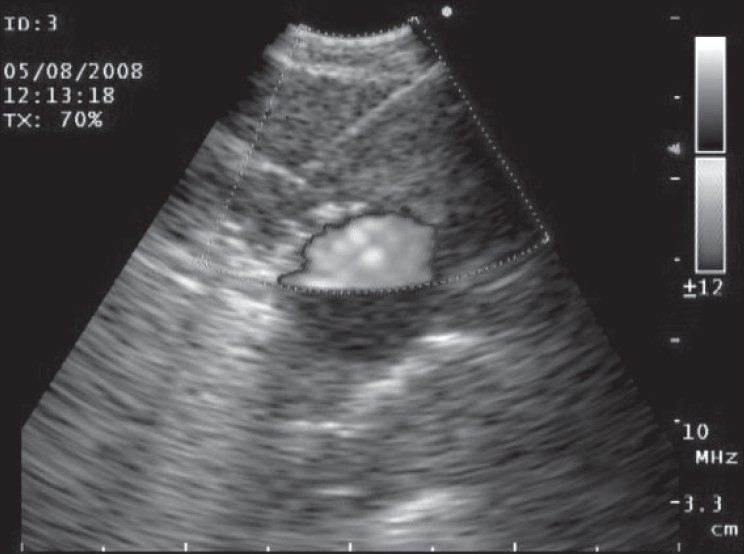
Real-time TBNA. Needle seen inside the lymph node. Doppler showing the blood vessels

Recently linear probe EBUS has been developed which allows real-time ultrasonic guidance during needle insertion [Figure 4 and Figure 5]. In a large series by Herth *et al*. real time EBUS-TBNA for enlarged mediastinal nodes on chest CT in suspected lung cancer patients, 572 lymph nodes were punctured and 535 (94%) resulted in diagnosis. Biopsies could be taken from all reachable lymph node stations (2L, 2R, 3, 4R, 4L, 7, 10R, 11R and 11L) and had a mean diameter of 1.6 cm. A sensitivity of 94%, a specificity of 100% and accuracy of 94% could be achieved. Similar results was found in other series also.^22^,^23^ EBUS-TBNA has been even found useful in sampling lymph nodes, which are not enlarged on chest CT. Herth *et al*. evaluated 100 lung cancer patients without enlarged lymph nodes.^24^ Surgical verification was performed in all patients. EBUS-TBNA had a sensitivity of 92% a specificity of 100% and a negative predictive value of 96%. Time required for performing EBUS-TBNA varies from 6.3 min to 30min as compared to conventional TBNA of 3.8 min.^6^,^15^,^21^ Recently EBUS-TBNA has been found to be a sensitive, specific and sufficiently accurate method to restage mediastinum after induction chemotherapy in patients with non-small cell lung cancer (NSCLC).^25^ However, a low negative predictive value necessitates surgical staging before deciding on thoracotomy.

EUS is also useful in assessing mediastinal lymphadenopathy. It has the added advantage of allowing to evaluate and do real time FNA of lymph node stations 8, 9, celiac axis lymph nodes and left adrenals which cannot be reached by EBUS and are occasional sites of lung cancer metastasis.^19^ A cross over trial by Herth FJ *et al*. concluded that both transesophageal and transbronchial ultrasound-guided FNAC have similar diagnostic yields, although transbronchial approach is superior for right-sided lymph nodes.^26^ By combining both procedures accuracy of more than 95% for mediastinal staging can be obtained.^27^ The terminology ‘medical mediastinoscopy’ has been introduced for the combined procedure (EUS-FNA + EBUS-TBNA) and it is expected that it will replace more invasive surgical mediastinoscopy for staging lung cancer.^28^

EBUS-TBNA can be used in the evaluation of mediastinal adenopathy due to other etiologies. EBUS-TBNA has a sensitivity of 85–91% for the primary diagnosis of sarcoidosis.^29^,^30^ Transbronchial lung biopsy (TBLB) which is the most commonly performed diagnostic procedure for sarcoidosis has a varying yield of 46–90% depending on the stage of sarcoidosis.^31^ Also TBLB is associated with 5% and 2% risk of bleeding and pneumothorax respectively, which EBUS-TBNA does not have.^32^

## COST EFFECTIVENESS OF EBUS

Although very useful in a variety of pulmonary diseases the question one needs to consider is whether EBUS is cost effective especially in a developing country like India. EBUS linear bronchoscope, processor, dedicated needles and radial probe add significantly to the cost of the procedure. But it has been found that EBUS-TBNA has significant cost savings.^33^ In a series of 108 lung cancer patients, 29 mediastinoscopies, four video-assisted thoracic surgery (VATS) procedures, eight thoracotomies and nine CT-guided lung biopsies were prevented due to EBUS-TBNA findings.^23^ Kunst P *et al*. after comparing the actual costs of 5 different strategies of mediastinal lymph node staging of lung cancer concluded that combined EBUS real time TBNA and conventional TBNA are the most cost-effective strategies.^34^ Recently Medical Services Advisory Committee of the Australian Government after reviewing the available evidence and economic analysis concluded that EBUS-guided procedures are cost saving for assessment of NSCLC and mediastinal/hilar masses, and for diagnosis of peripheral lung lesions when compared with current practices.^35^ Due to lack of clinical data an economic analysis was not conducted in the study done to assess airway involvement of endobronchial cancers with EBUS. The Committee recommended that public funding should be supported for EBUS-guided procedures for the staging of NSCLC.

Limited resources make the choice of decision between radial EBUS and linear EBUS difficult. Linear EBUS is an attractive option if one considers sampling mediastinal adenopathy since real-time guidance of needle is possible. However it is costlier and cannot be used for other indications of EBUS like assessing airway infiltration and localizing intrapulmonary lesions. There are no studies directly comparing the yield of radial EBUS and linear real time EBUS for mediastinal lesions. However for esophageal cancer a prospective trial has shown that there was no clinically relevant difference between curved array and radial echoendoscopy in the staging.^36^ So, one has to decide on the instrument depending on which indications one is likely to use EBUS more. More importantly before buying EBUS the pulmonologist has to remember that there has to be a high volume bronchoscopy list with reasonable number of patients with diagnosis of lung cancer. This is important to maintain competency, proficiency and gain experience. Also there needs to be a multidisciplinary team, which discusses and decides on the optimal management of the patient.

## CONCLUSIONS

Endobronchial ultrasound, which has been introduced about a decade ago, is becoming more and more popular. It is accurate, safe and is being used for an increasing number of indications. Radial probe EBUS can be used to evaluate airway invasion by tumors and analysis of peripheral pulmonary lesions. Linear EBUS provides real-time guidance for sampling mediastinal lesions. Although EBUS is very costly so far all the studies have shown that it is cost-effective. EBUS-TBNA and EUS-FNA can effectively replace mediastinoscopy as the preferred staging procedure for mediastinal lymph nodes in lung cancer patients. In India where tuberculosis is so prevalent it needs to be seen whether EBUS will mirror the high accuracy found in studies from developed countries.
